# Comparison of dextran and albumin on blood coagulation in patients undergoing major gynaecological surgery

**DOI:** 10.1186/s13741-018-0100-0

**Published:** 2018-09-06

**Authors:** Johann Sigurjonsson, David Hedman, Peter Bansch, Ulf Schött

**Affiliations:** 1 Department of Anaesthesia and Intensive Care, Institution of Clinical Science Lund, Medical Faculty, Lund University, Lund, Sweden; 2 Department of Anaesthesia and Intensive Care, Skåne University Hospital Lund, SE-221 85 Lund, Sweden

**Keywords:** Dextran, Albumin, Surgery, Blood-loss replacement, Fluid therapy, Gynaecologic cancer

## Abstract

**Background:**

Hydroxyethyl starches have been withdrawn from the European market. In Sweden, dextran was the main colloid until 2000, when starches overtook the market. After the recent 6S-trial, it was suggested that dextran could be reinstituted, but concerns for greater coagulopathy, bleeding and anaphylaxis still remain. An experimental study from our department indicated that isovolemic substitution of dextran-70 did not derange the von Willebrand function more than albumin 5%, considering the fact that dextran is hyperoncotic in comparison to albumin 5% and, therefore, induces a greater plasma volume expansion and thereby a greater dilutional coagulopathy.

**Methods:**

Eighteen patients undergoing major gynaecological surgery were assigned to receive either 5% albumin or 6% dextran-70 with 9 patients in each group. Standard coagulation tests, including prothrombin time (PT), activated partial thromboplastin time (aPTT), fibrinogen and platelet count, viscoelastic coagulation test thromboelastometry (ROTEM) and the Multiplate platelet aggregation test were used to test for coagulation defects at different time points perioperatively. Blood loss, blood loss replacement data and haemodynamic parameters were retrieved from anaesthetic and postoperative charts. A local departmental fluid and transfusion/infusion protocol assured haemoglobin > 90 g/l and mean arterial pressure > 65 mmHg with Ringer’s acetate in addition to the colloid use.

**Results:**

There were no differences in demographic data between the groups. The tissue factor-activated (EXTEM) clot-structure parameter ROTEM A10 was decreased significantly in the dextran group as compared to the albumin group after the infusion of 500 ml of either colloid solution. The PT and aPTT were significantly prolonged, and the platelet count decreased postoperatively in the dextran group, whereas albumin only deranged fibrinogen levels as compared to preoperative levels. There were no differences in Multiplate platelet aggregometry, amount of haemorrhage or transfusion of blood components between the groups.

**Conclusions:**

Standard plasma-based coagulation tests, platelet count and whole blood viscoelastic clot structure are affected by 6% dextran-70 to a greater extent than by 5% albumin, but platelet aggregation is not. Future studies should use more advanced haemodynamic monitoring to assess isovolemic plasma volume expansion with dextran and whether this affects haemostasis to a lesser degree.

## Background

Patients undergoing major gynaecological cancer resection surgery are at risk for fluid loss through evaporation, bleeding, capillary leakage and ascites formation. Crystalloids are often used for the initial fluid management; then colloids are added upon larger fluid/initial blood losses (Kristensen et al. 2014). The benefits of colloid infusion over crystalloid are still debated. A Cochrane review comparing the use of crystalloids and colloids in fluid resuscitation of critically ill patients did not show any reduction in mortality with the use of colloids. The authors argued that crystalloids should be used since they are less expensive (Perel et al. 2013). Still, colloids are often used for perioperative fluid therapy due to their better volume-expanding effects and better sustained volume effect with less risk for interstitial oedema affecting different organs (Vercueil et al. 2005). Another Cochrane review that compared the various colloid solutions available on the market showed no advantages of one over the other, in terms of safety and efficacy (Bunn and Trivedi 2012).

Dextran was the leading synthetic colloid in Sweden until 2001, but preference then shifted towards hydroxyethyl starch (HES) (Berseus et al. 2008). The disadvantages of dextran on haemostasis, an increased risk for anaphylaxis as compared to other synthetic colloids and the switch to low molecular weight heparins for thromboprophylaxis also contributed to this shift from dextran to starch.

There are only two recent randomised clinical studies on haemostasis and bleeding complications after perioperative dextran use (Rasmussen et al. 2015; Zdolsek et al. 2011).

A retrospective study on sepsis patients from Copenhagen, Denmark, showed greater bleeding (Hvidt and Perner 2012), while an older study on trauma patients did not indicate greater bleeding with the use of dextran (Modig 1986). A recent experimental study from our institution that focussed on comparing albumin with dextran during the isovolemic blood volume restitution of blood losses did not find any major differences in thromboelastometry (ROTEM) or von Willebrand factor analyses (Schött et al. 2017).

In a survey study, HES was the most used colloid in intensive care units in Scandinavia in 2008 (Perner et al. 2008). With growing concern regarding the safety of HES and its recent suspension from the market (Agency 2018), an increased use of albumin has been noted throughout Scandinavia (Kongsgaard et al. 2018). Albumin is relatively expensive but has shown increased survival in septic patients in a recent meta-analysis (Delaney et al. 2011). However, albumin has also been shown to impair coagulation and platelet function (De Jonge and Levi 2001; Li et al. 2015). Dextran has been used at our hospital, largely due to local routines and ongoing research in our experimental laboratory (Schött et al. 2017; Persson and Grände 2006; Dubniks et al. 2009).

The aim of this study was to see if there was any difference between fluid regimes with 6% dextran-70 or 5% albumin in major gynaecological surgery regarding routine coagulation tests, viscoelastic coagulation test ROTEM®, platelet function measured with a new bedside platelet aggrometer Multiplate®, bleeding or need for transfusion. We focused on patients undergoing surgery for ovarian cancer since this type of surgery is very standardised at our hospital, which leads to more comparable groups. They have advanced fluid shifts and are often hypovolemic, which is addressed in a recently finished study on albumin infusion rates to correct plasma volume deficits (Statkevicius et al. 2016). Also, since these patients often are hypercoagulable, surgeons were more positive to us, using dextran in comparison to albumin. Hypercoaguable patients probably have a better dilutive capacity before a colloid induced coagulopathy could endager perioperative haemostasis (Nilsson et al. 2016; Thomas et al. 2016).

## Methods

Ethical approval was obtained from the regional ethical review board (Lund, Protocol DNR 2010/482). Eighteen patients admitted to the University Hospital in Lund for elective gynaecological cancer operation between February 2014 and April 2014 were included in the study. All the patients’ informed and signed consent were retrieved before the operation.

The patients were anaesthetised in a standardised fashion. Fentanyl, propofol and rocuronium were used for induction, and sevoflurane was used to uphold anaesthesia. In cases where epidural analgesia was not used or was insufficient, fentanyl was given intraoperatively for pain management, and morphine was used for patient-controlled anaesthesia postoperatively.

Glucose 2.5% was used at a background infusion rate of 1 ml/kg/h intraoperatively and in the recovery ward as a routine in our hospital and is a part of the Air study protocol addressing the same type of patients (see above) (Statkevicius et al. 2016). Ringer’s acetate was infused at a basal rate of 4 ml/kg/h and could be increased if deemed necessary to maintain hourly diuresis at > 0.5 ml/kg of bodyweight and mean arterial pressure (MAP) of > 65 mmHg.

A local departmental fluid and transfusion protocol with packed red cells (PRBC) assured a haemoglobin > 90 g/l.

The colloid fluid was chosen by patient order—the first patient received albumin, the second dextran, the third albumin, and so on after consent approval from the patient and inclusion criteria were met. Either 5% albumin (CSL Behring, Germany) or 6% dextran (Macrodex®, Meda, Sweden) were used. Albumin was infused in consecutive batches of 250 ml and dextran in 500 ml batches. Dextran was restricted to 1000 ml to ensure patient haemostatic safety, as in vitro studies have indicated effects on coagulation and platelet function after only 500 ml (Aberg et al. 1979). Daily dextran infusions should be restricted to 500–1500 ml or 10–30 ml/kg of bodyweight (BW), according to the Pharmaceutical Specialties in Sweden (FASS). The FASS does not restrict albumin dosage according to haemostatic safety parameters, but it is well known that a dilutive coagulopathy and platelet dysfunction can be induced (De Jonge and Levi 2001). In vitro, this can be seen at 30% dilution (Nilsson et al. 2016), corresponding to a 1250 ml albumin in vivo infusion (calculated from female blood volume of 60 ml/kg BW and a median weight of 65 kg = 4000 ml; 30% dilution = 1200 ml). Therefore, we restricted the albumin dose to 1250 ml in this study.

The patients’ blood was sampled preoperatively (pre surgery), direct postoperatively (post surgery) and on the day following surgery (day 1). In addition, the blood was sampled after 500 ml colloid and 1000 ml colloid for ROTEM and Multiplate analyses.

The blood samples were drawn from an indwelling radial arterial catheter with a continuous flush and with a sampling membrane, which eliminated the need for disposing blood samples before sampling with the vacutainer technique.

The samples were analysed at each time point by conventional coagulation tests: prothrombin time (PT), activated partial thromboplastin time (aPTT), platelet count and fibrinogen, along with ROTEM and Multiplate. The blood samples for the ROTEM, aPTT, PT and fibrinogen tests were collected in 2 × 2.7 ml tubes containing 0.109 M citrate (BD, UK). The blood for the platelet counts was sampled in 3.0 ml EDTA-tubes. The blood for the Multiplate analyses was collected in 3.0 ml tubes containing recombinant hirudin (Dynabyte GmbH, Munich, Germany).

The PT, aPTT, fibrinogen and platelet count analyses were performed at an accredited hospital laboratory. The PT test was performed using a combined thromboplastin reagent (Stago prothrombin complex assay, SPA+, Stago). The Owren PT assay was calibrated using international normalised ratio (INR) calibrators certified by the Swedish external quality assessment organisation (Equalis, Uppsala, Sweden). The reference range for PT-INR is 0.9–1.2.

The aPTT was analysed with an aPTT reagent from Actin FSL (Siemens Healthcare Diagnostics). The reference range for aPTT has been established locally to 26–33 s. The plasma fibrinogen concentration was measured using the Dade Thrombin reagent (Siemens Healthcare Diagnostics, CS-5100). The local reference range for fibrinogen is set to 2–4 g/l.

The platelet counts were measured using the Sysmex XE 5000 cell counter (Sysmex Corp., Kobe, Japan). The locally determined reference range for platelet count is 165–387 × 10^9^/l for adult women.

The thromboelastometry was carried out using the rotational thromboelastometry (ROTEM®; TEM Innovations GmbH, Germany) instrument according to the manufacturer’s instructions. The ROTEM assays were run for 60 min, and samples were analysed with two different ROTEM tests: EXTEM and FIBTEM. The variables that were registered for EXTEM (normal range within brackets) were CT/clotting time (42–74 s), CFT/clot formation time (46–148 s), AA/alpha-angle (63–81°), A10/clot formation at 10 min (43–65 mm) and MCF/maximum clot formation (63–81 mm). For FIBTEM, A10 (9–24 mm) and MCF (9–25 mm) were analysed.

The impedance aggregometry was carried out using the Multiplate® analyser (Roche Diagnostics, Basel, Switzerland) according to the manufacturer’s instructions. Three platelet receptor agonists were used: adenosin phosphate (ADP), collagen (COL) and a thrombin receptor activating peptide (TRAP), and area under the curve (AUC) were measured (normal range within brackets): ADP-test (53–122), COL-test (46–117) and TRAP-test (94–156).

The statistical analyses were performed using Graphpad Prism 6. The Wilcoxon rank-sum test was used to calculate the changes in coagulation parameters by comparing the post 500 ml colloids, post 1000 ml colloids and post surgery and day 1 values to the control pre surgery value (only for dextran due to inadequate patient numbers receiving 1000 ml albumin infusions; *n* = 2). The Mann-Whitney U test was used to calculate the changes between dextran and albumin groups. Due to multiple statistical calculations, the *p*-values are presented after Bonferroni correction. *p* < 0.05 was considered significant. The median values are given, and the results are presented as tables and boxplots. The data was not regarded as normal distributed.

## Results

Eighteen patients were included in this study. All had ovarian cancer in advanced stage; therefore, surgery involved ovarian resection, hysterectomy and lymph node removals. Seven patients received dextran, and seven received albumin. Four patients did not require volume support with colloids. Calculating statistical power from 14 to 18 patients and dilutive effects on fibrinogen and differences between albumin and dextran on ROTEM A10 indicates that our pilot study is underpowered at a 0.65–0.75 level. There were no significant differences in age, bleeding or duration of surgery; amount of fluids, colloids or units (250 ml) of PRBC given; or numbers of postoperative complications (Table 1).

**Table 1:** Demographics of the two different groups. No significant differences (*p* > 0.05) between the dextran and albumin substitution groups. Packed red blood cells (PRBCs)

	Dextran *n* = 7	Albumin *n* = 7	*p* value
Age			
(Average)	61	62.9	0.7806
(Median)	59	65	
(Min-Max)	(54–78)	(45–77)	
Bleeding during surgery (ml)			
(Average)	1110	679	0.6953
(Median)	900	750	
(Min-Max)	(200–3500)	(200–1100)	
Surgery time (minutes)			
(Average)	281	325	0.3759
(Median)	305	358	
(Min-Max)	(162–369)	(133–411)	
Total amount of fluid given (ml)			
(Average)	4350	5040	0.9656
(Median)	4580	4270	
(Min-Max)	(2400–6950)	(2260–8940)	
Total amount of colloids given (ml)			
(Average)	982	821	0.6253
(Median)	1000	750	
(Min-Max)	(500–1750)	(250–1250)	
Total amount of PRBCs given (units)			
(Average)	1.57	0.43	0.4126
(Median)	0	0	
(Min-Max)	(0–6)	(0–2)	
Number of patients with postoperative complications	5	6	–

**Fig. 1:**
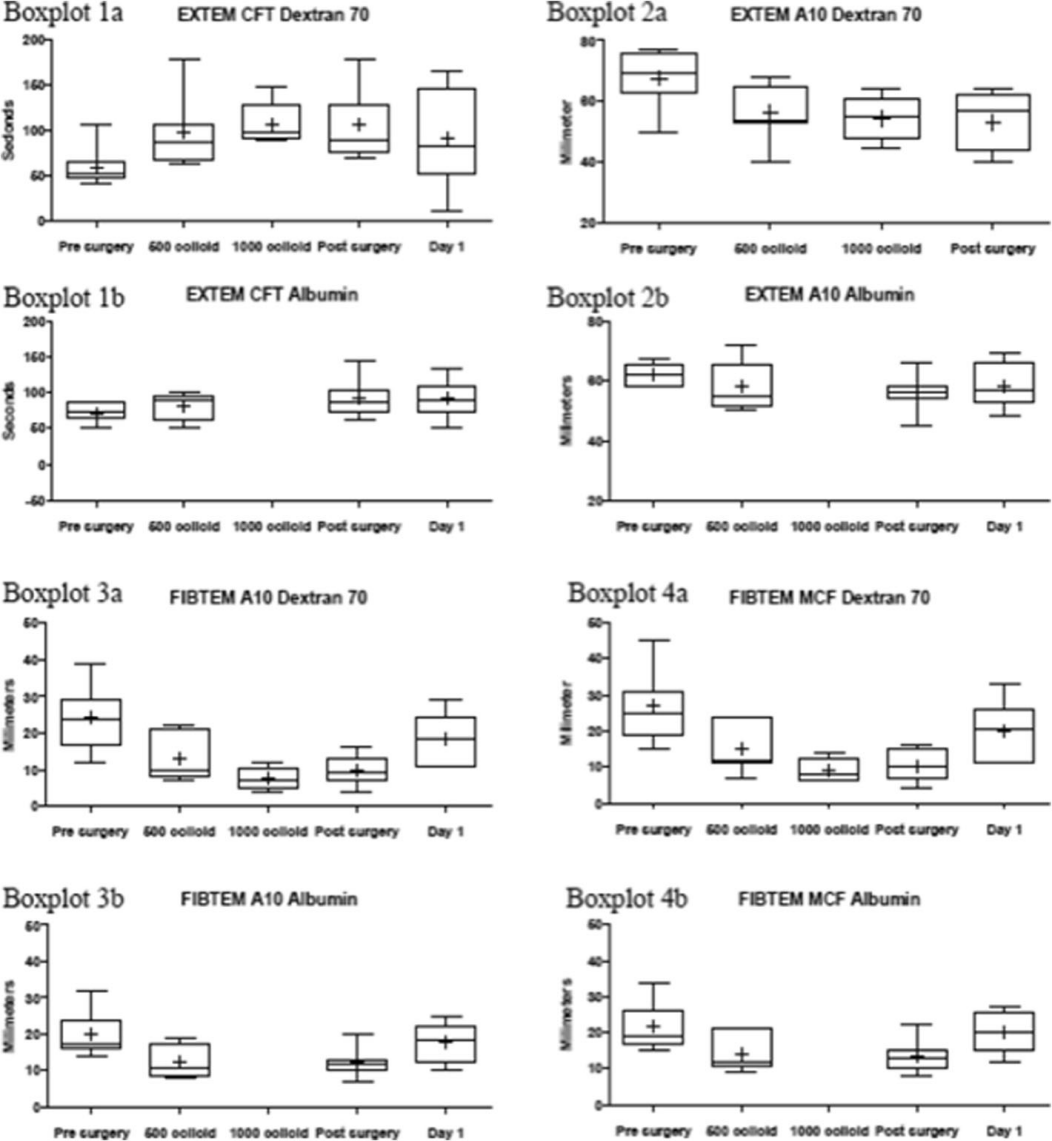
Sample figure describing the different ROTEM values. Boxplot 1 a + b: Change of EXTEM CFT values over time for dextran and albumin. Albumin 1000 colloid omitted due to limited data (*n* = 2). Boxplot 2 a + b: Change of EXTEM A10 values changes over time for dextran and albumin. Albumin 1000 colloid omitted due to limited data (*n* = 2). Boxplot 3 a + b: Change of FIBTEM A10 values over time for dextran and albumin. Albumin 1000 colloid omitted due to limited data (*n* = 2). Boxplot 4 a + b: Change in FIBTEM MCF values over time for dextran and albumin. Albumin 1000 colloid omitted due to limited data (*n* = 2)

The ROTEM EXTEM intragroup changes for both groups are presented in Fig. 1. There were no significant changes (*p* < 0.05). For the dextran group, the CFT increased from 51 s (s) pre surgery to 86 s after infusion of 500 ml of dextran and increased to 89 s post surgery. The alpha-angle decreased from 80° pre surgery to 73° after infusion of 500 ml of dextran. The EXTEM A10 decreased from a pre-surgery value of 69 mm (mm) to 54 mm after infusion of 500 ml of dextran. The MCF decreased from 74 mm pre surgery to 65 mm after infusion of 500 ml of dextran. For the albumin group, the CFT increased from 72 s pre surgery to 92 s post surgery. The A10 fell from 62 s pre surgery to 56 s post surgery.

The FIBTEM A10 and MCF intragroup changes for dextran and albumin are also presented in Fig. 1. There were no significant changes (*p* < 0.05). For the dextran group, the FIBTEM A10 fell from 24 mm pre surgery to 10 mm after infusion of 500 ml of dextran and to 9 mm post surgery. The MCF fell from 25 mm pre surgery to 12 mm after infusion of 500 ml of dextran and to 10 mm post surgery. For the albumin group, the A10 fell from 17 mm pre surgery to 11 mm after infusion of 500 ml of albumin. The MCF fell from 19 mm pre surgery to 12 mm after infusion of 500 ml of albumin.

The changes over time for the two groups, expressed as Δ values for A10 and MCF (EXTEM + FIBTEM), are presented in Table 2. A significant intergroup difference (*; *p* < 0.05) between pre surgery and after the infusion of 500 ml of dextran was seen for EXTEM A10 (*p* = 0.0456), and the difference remained almost significant until post surgery (*p* = 0.0594).

**Table 2:** Delta values (Δ) (median) describing the change compared to pre surgery in EXTEM and FIBTEM A10—clot width at 10 min and MCF—maximum clot formation for dextran and albumin at the different time points.* *p* < 0.05 was calculated using Mann-Whitney U test with Bonferroni correction for intergroup comparison

	Dextran	Albumin	*p* value
EXTEM A10			
Δ Pre surgery and 500 ml colloid	10	4.5	0.0456*
Δ Pre surgery and post Surgery	15	5	0.0594
Δ Pre surgery and day 1	9	5	0.4545
EXTEM MCF			
Δ Pre surgery and 500 ml colloid	7	3.5	0.2379
Δ Pre surgery and post surgery	11	5	0.1503
Δ Pre surgery and day 1	7.5	3.5	0.2598
FIBTEM A10			
Δ Pre surgery and 500 ml colloid	8	6.5	0.5943
Δ Pre surgery and post surgery	15	6	0.3549
Δ Pre surgery and day 1	7	1	0.6658
FIBTEM MCF			
Δ Pre surgery and 500 ml colloid	8	6.5	0.369
Δ Pre surgery and post surgery	14	7	0.1206
Δ Pre surgery and day 1	7.5	1.5	0.4806

There were no significant differences (*p* < 0.05) in the Multiplate AUC values for either of the groups (Table 3).

**Table 3:** Multiplate values

Dextran Multiplate	Pre surgery *n* = 7	500 colloid *n* = 7	1000 colloids *n* = 5	Post surgery *n* = 7	Day 1 *n* = 6
ADP (AUC)					
(Average)	89.1	76.6	95.8	81.9	77.2
(Median)	86	87	96	84	75
(Min-Max)	(42–128)	(44–110)	(70–118)	(44–108)	(38–106)
COL (AUC)					
(Average)	81.7	77	99.2	85.1	82.2
(Median)	84	62	95	82	74
(Min-Max)	(41–130)	(40–118)	(82–121)	(38–123)	(60–121)
TRAP (AUC)					
(Average)	118.7	125.4	138	128.4	107.8
(Median)	117	124	131	130	108.5
(Min-Max)	(67–157)	(78–170)	(106–168)	(87–171)	(80–134)
Albumin Multiplate	Pre surgery *n* = 7	500 colloid *n* = 6	1000 colloids *n* = 2	Post surgery *n* = 7	Day 1 *n* = 6
ADP (AUC)					
(Average)	88.3	93	52.5	84.7	72.2
(Median)	89	83.5	52.5	86	82
(Min-Max)	(63–116)	(67–124)	(22–83)	(36–124)	(32–99)
COL (AUC)					
(Average)	83.7	97.7	63	86.9	77
(Median)	78	82	63	88	85
(Min-Max)	(69–103)	(60–149)	(38–88)	(43–149)	(38–107)
TRAP (AUC)					
(Average)	132.4	124.7	84.5	123.6	102.2
(Median)	137	120.5	84.5	126	88
(Min-Max)	(113–162)	(85–176)	(56–113)	(76–176)	66–143)

There were no significant intragroup changes (*p* > 0.05)

*ADP* adenosin phophate, *COL* collagen, *TRAP* thrombin receptor activating peptide for dextran and albumin at the different time points

The changes in routine coagulation values for both groups are presented in Table 4. In the dextran group, the platelet count significantly decreased from 354 × 10^9^/l pre surgery to 317 × 10^9^/l (*p* = 0.0312) post surgery and to 221 × 10^9^/l (*p* = 0.0312) on day one. The PT-INR increased from 1.0 pre surgery to 1.2 (*p* = 0.0312) post surgery to 1.3 (*p* = 0.0312) on day one, and the aPTT changed from 25 s pre surgery to 31 s (*p* = 0.0312) post surgery and to 30 s (*p* = 0.0312) on day one. The fibrinogen changed from 4.2 g/l pre surgery to 1.9 g/l (*p* = 0.0312) post surgery.

**Table 4:** Standard laboratory (lab) values for Dextran and Albumin at the different time points

	Pre surgery *n* = 7	Post surgery *n* = 7	Day 1 *n* = 6
Dextran routine lab			
Platelet count			
(Average)	333.9	280*	218.8
(Median)	354	317	221
(Min-Max)	(160–466)	(114–432)	(83–368)
PT-INR			
(Average)	0.99	1.3*	1.3*
(Median)	1	1.2	1.3
(Min-Max)	0.9 1	(1.1–1.7)	(1.1–1.5)
aPTT			
(Average)	25.6	36.6*	31.6*
(Median)	25	31	30
(Min-Max)	(24–28)	(27–61)	(28–42)
Fibrinogen			
(Average)	4.6	2.0*	3.3
(Median)	4.2	1.9	3.35
(Min-Max)	(3.2–8.2)	(1–3)	(1.6–5.1)
Albumin routine lab			
Platelet count			
(Average)	328.4	267.7	228.4*
(Median)	277	241	188
(Min-Max)	(238–483)	(143–422)	(124–381)
PT-INR			
(Average)	1	1.3	1.3*
(Median)	1	1.3	1.4
(Min-Max)	(1–1)	(1–1.5)	(1.1–1.5)
aPTT			
(Average)	27.1	30.7	32.3
(Median)	27	29	33
(Min-Max)	(25–29)	(25–41)	(26–37)
Fibrinogen			
(Average)	4	2.1*	3.3
(Median)	4	1.8	3.2
(Min-Max)	(2.9–5.4)	(1.4–4.2)	(2.5–4.2)

*Significant (*p* < 0.05) intragroup change from the respective pre surgery value

In the albumin group, the platelet count decreased from 328 × 10^9^/l pre surgery to 118 × 10^9^/l (*p* = 0.0312) on day one. The PT-INR was prolonged from 1.0 pre surgery to 1.3 (*p* = 0.0312) on day one. The fibrinogen decreased from 4.0 g/l pre surgery to 2.1 g/l (*p* = 0.0312) post surgery.

## Discussion

Signs of impaired coagulation were seen already after infusion of 500 ml of dextran when measured with ROTEM and traditional coagulation tests. Statistically significant changes in the routine laboratory values were observed in both groups. The dextran group showed significant intragroup decreases in both platelet counts and fibrinogen levels, as well as in the PT-INR and aPTT, measured pre surgery, post surgery and on day one after surgery.

The albumin group showed similar tendencies, though first statistically significant on day one post surgery. There were no significant differences between the groups in bleeding, amount of administered crystalloids, colloids or blood products or numbers of postoperative complications. When valuing the changes over time, presented as Δ values and illustrated in Table 2, a significant decrease in EXTEM A10 was observed after 500 ml infusion in the dextran group, whereas the decrease in the albumin group was much more discrete and did not reach statistical significance.

Our finds corroborate with those of Rasmussen et al. finding a greater dextran induced dilutional effect on TEG clot structure/platelet function than with Ringer’s lactate during radical cystectomy (Rasmussen et al. 2015). The decrease in TEG maximal amplitude (MA) corresponding to ROTEM MCF correlated to more bleeding, contradicting our finds. An isooncotic 3% dextran-60 solution (Plasmadex®) + PRBC to replace blood loss during hip replacement up to 50% of the calculated blood volume did not increase bleeding as compared to whole blood (Schött et al. 1985). However, hypertonic saline with dextran (RescueFlow®) induced more hypocoagulation and hyperfibrinolysis than hypertonic saline without dextran in trauma patients (Delano et al. 2015). The same was seen for cardiac surgery patients (Bueno et al. 2004).

There are few prospective clinical studies on dextran as a plasmasubstitute, comparing it to other colloids. Zdolsek et al. compared different types of HES with 6% dextran 70 (Zdolsek et al. 2011). They found better perioperative haemodynamics with dextran, but no major coagulation, bleeding, or transfusion differences between the colloids. The dextran dilutive effect on ROTEM clot structure endured longer postoperatively (not significantly lower as compared to HES) probably related to its longer half-time and plasma volume expanding effect.

Halonen et al. compared HES, dextran 70 and 4% albumin (all 20 ml/kg) during abdominal surgery for initial haemodilution and then whole blood for further blood loss replacement (Halonen et al. 1987a). A dilutional effect on serum albumin and prothrombin-proconvertin level was observed during the day of surgery for all the colloids. On the first postoperative day, these laboratory parameters were equally low for all colloids. Platelet counts and bleeding times were adequate. Partial thromboplastic time values were unchanged. The factor VIII procoagulant, related antigen (R:Ag, vWF:Ag) and ristocetin cofactor levels (high preoperatively) also remained at an adequate level. There were no increased bleeding in the dextran group. However, non-steroidal anti-inflammatory drugs increased bleeding tendency together with dextran (Halonen et al. 1987b).

The ROTEM FIBTEM analysis is not optimally platelet inhibited so the signal can still be affected by platelets (Schöchl et al. 2011), probably more in the later stages of the fibrin polymerisation. This could explain the difference in between A10 and MCF as the platelet interaction is a late phenomena strengthening the clot structure whereas the A10 reflects early polymerisation. Dextran effects on platelets are maximal first after 6 h (Aberg et al. 1979). Fibrin polymerisation is complex and other factors than colloids, like smoking, genetics, tumour induced hypercoaguabilty, atherosclerosis and medications can be involved (Weisel and Litvinov 2013).

The measurements illustrated in Fig. 1 show a decrease in the FIBTEM A10 and MCF in both groups. Two patients in the dextran group and one patient in the albumin group had values of A10 < 7 mm and MCF < 10 mm, which are often regarded as trigger values for corrective treatment with plasma or fibrinogen concentrate in transfusion protocols using ROTEM (Bolliger et al. 2012). Fibrinogen concentrate appears to reverse the FIBTEM defects more potently after albumin dilution than after dextran dilution in vitro (Winstedt et al. 2013). No patients in our study received fibrinogen concentrate. Being an acute phase reactant, fibrinogen plasma levels start to recover immediately after bleeding and colloid infusions have stopped (Nilsson et al. 2016; Thomas et al. 2016; Wyatt et al. 2013). This can be seen in Table 4, with normalised fibrinogen levels at day 1.

The recent trend shift in colloid consumption following a dramatic reduction in HES sales has brought other solutions back into the limelight. Albumin use has increased steadily in the Nordic countries since 2012, while dextran has seen sales drop by more than 40% over the same period (Kongsgaard et al. 2018).

As mentioned earlier, dextran is known to impair coagulation through various mechanisms, with a risk of bleeding. Dextran induces the so-called von Willebrand-syndrome, which signifies a decrease in the levels/activity of the von Willebrand factor (vWF) as well as factor VIII (Aberg et al. 1979; Batlle et al. 1985). The mechanism behind this phenomenon is not fully understood and has even been observed with other artificial colloids. One theory is that macromolecules present in colloids bind to vWF, thus, accelerating its elimination (Treib et al. 1997; de Jonge et al. 1998). Additionally, dextran affects fibrinogen polymerisation by co-binding, increases the circulating levels of tissue type plasminogen activator (t-PA) and decreases the levels of fibrinolysis plasminogen activator inhibitor-1 (PAI-1) (Eriksson and Saldeen 1995). This, in turn, leads to weaker fibrinogen-polymerisation, thus making the clot more prone to lysis (Carlin et al. 1979).

Our group recently compared the effect of resuscitation with either 6% dextran-70 or 5% albumin in equipotent doses regarding plasma volume expansion on coagulation in a guinea pig haemorrhagic model. In that study, albumin reduced vWF levels to a larger extent than dextran, both immediately after resuscitation and 4 h later, but no difference in p-vWF GP1bA activity or in plasma fibrinogen levels could be detected (Schött et al. 2017). Dextran-70 resulted in a transient prolongation of thromboelastography (TEG) kinetics (K corresponding to ROTEM CFT) at the completion of resuscitation compared to albumin. The TEG reaction time (R, corresponding to ROTEM CT) and maximal amplitude (MA corresponding to ROTEM MCF) did not differ between the treatments at any of the time points (Schött et al. 2017). In vitro studies comparing dextran with other colloids indicated a deterioration in clot structure measured with another technique than ROTEM/TEG (Carr and Gabriel 1980; Carr 1986).

A recently published propensity-score matching study to evaluate the effect of dextran-70 on organ failure in severe sepsis did not show any signs of higher incidence of severe bleeding with dextran. Neither did it provide any evidence for detrimental effects of dextran-70 on kidney function, need for organ support or mortality (Bentzer et al. 2017).

Albumin is an endogenous protein with a molecular size of 39 kD. Albumin is the main component responsible for the colloid osmotic pressure in plasma and plays an important role in preserving intravascular volume (Statkevicius et al. 2016). Infusion with albumin derived from human plasma is frequently used for plasma volume expansion in the perioperative and intensive care settings. Albumin is an important transport protein and a free radical scavenger (Mortimer et al. 2014). Since albumin is derived from human plasma, it is relatively expensive and may facilitate transmission of infectious diseases. Albumin can also induce a dilutional coagulopathy and platelet defects (De Jonge and Levi 2001). Recent research has focussed on the glycocalyx, the vascular endothelium, and the role of plasma in maintaining its integrity. Albumin has, among other plasma proteins, been reported to have a protective effect on the glycocalyx (Schött et al. 2016). It has been speculated that the decrease in plasma concentration of markers of glycocalyx degradation after resuscitation with plasma as compared to crystalloids are largely secondary to differences in plasma volume and may not accurately reflect effects of FFP on the glycocalyx (Nelson et al. 2016).

We did not find any significant differences in the Multiplate AUC values for either of the groups. A similar find was detected with in vitro dilution with dextran 40 (Kam et al. 2017).

The Multiplate device measures platelet function using different activating factors corresponding to platelet receptors and has similar limitations as ROTEM. The Multiplate, therefore, cannot measure platelet dysfunction caused by vWF defects (Schött and Johansson 2013). Thus, in our case, we may suspect that the Multiplate failed to reflect a platelet dysfunction as dextran has a known effect on vWF. Studies have found that platelet counts of < 150 × 10^9^/l may influence the Multiplate, which should be considered in clinical settings (Stissing et al. 2011). The platelet count decreased more in the dextran group in the present study, but the Multiplate did not detect any differences between the albumin and dextran patients. In an earlier study on elective neurosurgery brain tumour resection from our centre, albumin decreased Multiplate aggregation more than HES (Li et al. 2015). An in vitro study with extreme colloid dilution (gelatine or HES; 60%) decreased Multiplate aggregation (Kind et al. 2013). Neither of these studies measured platelet counts. In fact, in the two patients receiving 1000 ml of albumin 5%, Multiplate aggregation was lower than in the six patients receiving 1000 ml of dextran-70 (Table 2). Whether this is a true effect of the colloid or is related to lower platelet counts needs to be studied with more patients. Currently, the European Society of Anaesthesiologists guidelines do not recommend perioperative Multiplate monitoring other than in patients with platelet inhibiting drugs (Kozek-Langenecker et al. 2014).

There were no statistically significant differences in bleeding or blood transfusions between the dextran or albumin groups, although the albumin group had less median bleeding (750 ml) as compared to the dextran group (900 ml) and needed fewer transfusions of PRBC. The low number of patients receiving colloids in the present study (*n* = 14 in total) and the variation in preoperative coagulation profiles—especially preoperative ROTEM indicating hyper-, normo- and hypocoagulation (Table 2, Fig. 1)—blur the interpretation of whether it is the colloid-induced coagulopathy, the difficulties in resection of tumours or the difficulties in obtaining surgical haemostasis that increases bleeding and the higher transfusion rate in the dextran group. The large ranges in bleeding (200–3500 ml in the dextran group and 200–1100 ml in the albumin group) are also a confounding factor impeding the interpretation of our results. A previous study from our centre has implemented the nomenclature *dilutional capacity* to refer to the above-described preoperative coagulation strength measured with ROTEM and specific coagulation factor analyses (fibrinogen, FXIII activity, thrombin-antithrombin complex [TAT] and plasmin-α2-antiplasmin complex [PAP]) to individualise the colloid use to avoid colloid induced dilutional coagulopathies (Nilsson et al. 2016). This approach should be tested, but it requires at least a preoperative ROTEM/TEG testing, currently not recommended by the European Society of Anaesthesiologists (ESA) guidelines for perioperative bleeding management (Kozek-Langenecker et al. 2014).

Perioperative TEG hypercoagulation has also been suggested to reflect the degree of malignancy of ovarian masses (Amirkhosravi et al. 2013). Dextran has been suggested to reduce thromboembolism in cancer surgery (Foster et al. 2017). Dextrans have also been used in vascular surgery to diminish the risk of stroke associated with carotid endarterectomy and in “difficult” infragenicular lower extremity bypasses (Abir et al. 2004). However, a recent retrospective study found no reduction in perioperative stroke, but more cardiac failure and myocardial infarction after dextran (Farber et al. 2013).

### Limitations

There were several limitations in this study. It was an open observational screening study of coagulation changes with two fluid regimes, and it was non-randomised and not blinded. It was a pilot study of a problematic group of patients undergoing major gynaecological cancer surgery that had a risk for major bleeding and often showed rapid fluid shifts intra- and postoperatively that demanded a high level of postoperative surveillance. It was underpowered to detect a correlation between ROTEM, laboratory and Multiplate analyses and clinical parameters such as bleeding, the need for transfusions and postoperative complications. The ROTEM or Multiplate system results might not always correlate with clinical bleeding, as there were no blood flow/shear stress, effects of endothelial activation/inhibition or effects of the glycocalyx layer that interacted with coagulation and platelet activation in vivo. The study also had a small sample size, reflecting the limited period of patient recruitment time, but our aim is to proceed with this study up to 60 patients.

## Conclusion

Standard plasma-based coagulation tests, platelet count and whole blood viscoelastic clot structure are affected by 6% dextran-70 to a greater extent than by 5% albumin, but platelet aggregation is not. Future studies should use more advanced haemodynamic monitoring to assess isovolemic plasma volume expansion with dextran and whether this affects haemostasis to a lesser degree.

## Abbreviations

AUC: Area under the curve
6S: Scandinavian Starch for Severe Sepsis/Septic Shock trial
A10: Clot formation at 10 min
AA: Alpha-angle
ADP: Adenosin phosphate
aPTT: Activated partial thromboplastin time
BW: Body weight
CFT: Clot formation time
COL: Collagen
CT: Clotting time
F: Factor
FASS: Pharmaceutical Specialties in Sweden
HES: Hydroxyethyl starch
INR: International normalised ratio
K: Kinetics
kD: kiloDalton
MA: Maximal amplitude
MAP: Mean arterial pressure
MCF: Maximum clot formation
PAI-I: Plasminogen activator inhibitor-1
PAP: Plasmin-α2-antiplasmin complex
POC: Point of care
PRBC: Packed red blood cells
PT: Prothrombin time
R: Reaction time
ROTEM: Thromboelastometry
TAT: Thrombin-antithrombin complex
TEG: Thromboelastography
t-PA: Tissue type plasminogen activator
TRAP: Thrombin receptor activating peptide
vWF: von Willebrand factor

## References

[CR1] Aberg M, Hedner U, Bergentz SE (1979). Effect of dextran on factor VIII (antihemophilic factor) and platelet function. Ann Surg.

[CR2] Abir F, Barkhordarian S, Sumpio BE (2004). Efficacy of dextran solutions in vascular surgery. Vasc Endovasc Surg.

[CR3] Agency EM (2018). Hydroxyethyl-starch solutions for infusion to be suspended – CMDh endorses PRAC recommendation suspension due to serious risks of kidney injury and death in certain patient.

[CR4] Amirkhosravi A, Bigsby G, Desai H, Rivera-Amaya M, Coll E, Robles-Carrillo L (2013). Blood clotting activation analysis for preoperative differentiation of benign versus malignant ovarian masses. Blood Coagul Fibrinolysis.

[CR5] Batlle J, Del Rio F, Fernandez L (1985). Effect of dextran on factor VIII/von Willebrand factor structure and function. Thromb Haemost.

[CR6] Bentzer P, Broman M, Kander T (2017). Effect of dextran-70 on outcome in severe sepsis; a propensity-score matching study. Scand J Trauma Resusc Emerg Med.

[CR7] Berseus O, Norda R, Safwenberg J (2008). Blodverksamheten i Sverige 2007: omfattning, kvalitet och säkerhet.

[CR8] Bolliger D, Seeberger MD, Tanaka KA (2012). Principles and practice of thromboelastography in clinical coagulation management and transfusion practice. Transfus Med Rev.

[CR9] Bueno R, Resende AC, Melo R (2004). Effects of hypertonic saline-dextran solution in cardiac valve surgery with cardiopulmonary bypass. Ann Thorac Surg.

[CR10] Bunn F, Trivedi D (2012). Colloid solutions for fluid resuscitation. Cochrane Database Syst Rev.

[CR11] Carlin G, Modig J, Saldeen T (1979). Effect of infusion of dextran 70 on fibrinolysis inhibition activity in human serum. Acta Chir Scand.

[CR12] Carr ME (1986). Effect of hydroxyethyl starch on the structure of thrombin- andreptilase-induced fibrin gels. J Lab Clin Med.

[CR13] Carr ME, Gabriel DA (1980). The effect of dextran 70 on the structure of plasma-derived fibrin gels. J Lab Clin Med.

[CR14] De Jonge E, Levi M (2001). Effects of different plasma substitutes on blood coagulation: a comparative review. Crit Care Med.

[CR15] de Jonge E, Levi M, Berends F, van der Ende AE, ten Cate JW, Stoutenbeek CP (1998). Impaired haemostasis by intravenous administration of a gelatin-based plasma expander in human subjects. Thromb Haemost.

[CR16] Delaney AP, Dan A, McCaffrey J, Finfer S (2011). The role of albumin as a resuscitation fluid for patients with sepsis: a systematic review and meta-analysis. Crit Care Med.

[CR17] Delano MJ, Rizoli SB, Rhind SG, Cuschieri J, Junger W, Baker AJ, Dubick MA, Hoyt DB, Bulger EM (2015). Prehospital resuscitation of traumatic hemorrhagic shock with hypertonic solutions worsens hypocoagulation and hyperfibrinolysis. Shock.

[CR18] Dubniks M, Persson J, Grände PO (2009). Comparison of the plasma volume-expanding effects of 6% dextran 70, 5% albumin, and 6% HES 130/0.4 after hemorrhage in the guinea pig. J Trauma - Inj Infect Crit Care.

[CR19] Eriksson M, Saldeen T (1995). Effect of dextran on plasma tissue plasminogen activator (t-PA) and plasminogen activator inhibitor-1 (PAI-1) during surgery. Acta Anaesthesiol Scand.

[CR20] Farber A, Tan TW, Rybin D, Kalish JA, Hamburg NM, Doros G, Goodney PP, Cronenwett JL, Vascular Study Group of New England (2013). Intraoperative use of dextran is associated with cardiac complications after carotid endarterectomy. J Vasc Surg.

[CR21] Foster JM, Sleightholm R, Watley D, Wahlmeier S, Patel A (2017). The efficacy of dextran-40 as a venous thromboembolism prophylaxis strategy in cytoreductive surgery and hyperthermic intraperitoneal chemotherapy. Am Surg.

[CR22] Halonen P, Linko K, Myllylä G (1987). A study of haemostasis following the use of high doses of hydroxyethyl starch 120 and dextran in major laparotomies. Acta Anaesthesiol Scand.

[CR23] Halonen P, Linko K, Wirtavuori K, Hästbacka J, Ikkala E (1987). Evaluation of risk factors in intraoperative bleeding tendency. Ann Chir Gynaecol.

[CR24] Hvidt LN, Perner A (2012). High dosage of dextran 70 is associated with severe bleeding in patients admitted to the intensive care unit for septic shock. Dan Med J.

[CR25] Kam P, Liou J, Yang K (2017). In vitro evaluation of the effect of haemodilution with dextran 40 on coagulation profile as measured by thromboelastometry and multiple electrode aggregometry. Anaesth Intensive Care.

[CR26] Kind SL, Spahn-Nett GH, Emmert MY, Eismon J, Seifert B, Spahn DR (2013). Is dilutional coagulopathy induced by different colloids reversible by replacement of fibrinogen and factor xiii concentrates?. Anesth Analg.

[CR27] Kongsgaard UE, Holtan A, Perner A (2018). Changes in colloid solution sales in Nordic countries. Acta Anaesthesiol Scand.

[CR28] Kozek-Langenecker SA, Imberger G, Rahe-Meyer N, Afshari A, European Society of Anaesthesiology Guidelines Task Force (2014). Reply to: ESA guidelines on the management of severe perioperative bleeding. Eur J Anaesthesiol.

[CR29] Kristensen SD, Knuuti J, Saraste A, Anker S, Bøtker HE, De Hert S (2014). 2014 ESC/ESA guidelines on non-cardiac surgery. Eur J Anaesthesiol.

[CR30] Li N, Statkevicius S, Asgeirsson B, Schött U (2015). Effects of different colloid infusions on ROTEM and Multiplate during elective brain tumour neurosurgery. Perioper Med (Lond).

[CR31] Modig J (1986). Effectiveness of dextran 70 versus Ringer’s acetate in traumatic shock and adult respiratory distress syndrome. Crit Care Med.

[CR32] Mortimer GM, Butcher NJ, Musumeci AW, Deng ZJ, Martin DJ, Minchin RF (2014). Cryptic epitopes of albumin determine mononuclear phagocyte system clearance of nanomaterials. ACS Nano.

[CR33] Nelson A, Statkevicius S, Schött U, Jophanssaon PI, Bentzer P. Effects of fresh frozen plasma, Ringers´s acetate and albumin omn plasma volume and on circulating glycocalyx components following haemorrhagic shock in rats. Intensive Care Med Exp. 2016;4:6. 10.1186/s40635-016-0080-7 PMC4777969 26940500

[CR34] Nilsson CU, Strandberg K, Engström M, Reinstrup P (2016). Coagulation during elective neurosurgery with hydroxyethyl starch fluid therapy: an observational study with thromboelastometry, fibrinogen and factor XIII. Perioper Med (Lond).

[CR35] Perel P, Roberts I, Ker K. Colloids versus crystalloids for fluid resuscitation in critically ill patients. Cochrane Database Syst Rev. 2013;(2):CD000567. 10.1002/14651858.CD000567.pub6 23450531

[CR36] Perner A, Åneman A, Guttormsen AB, Kárason S, Tenhunen J (2008). Preferences for colloid use in Scandinavian intensive care units. Acta Anaesthesiol Scand.

[CR37] Persson J, Grände PO (2006). Plasma volume expansion and transcapillary fluid exchange in skeletal muscle of albumin, dextran, gelatin, hydroxyethyl starch, and saline after trauma in the cat. Crit Care Med.

[CR38] Rasmussen KC, Hoejskov M, Johansson PI, Kridina I, Kistorp T, Salling L, Nielsen HB, Ruhnau B, Pedersen T, Secher NH (2015). Coagulation competence for predicting perioperative hemorrhage in patients treated with lactated Ringer's vs. dextran - randomized controlled trial. BMC Anesthesiol.

[CR39] Schöchl H, Cotton B, Inaba K, Nienaber U, Fischer H, Voelckel W, Solomon C (2011). FIBTEM provides early prediction of massive transfusion in trauma. Crit Care.

[CR40] Schött U, Johansson PI (2013). Bringing flow into haemostasis diagnostics. Br J Anaesth.

[CR41] Schött U, Kander T, Bentzer P. Effects of dextran-70 and albumin on coagulation in expewrimental hemorrhage in the guinea pig. Shock. 2017;18. [Epub ahead of print]. 10.1097/SHK.0000000000001025 PMC6072367 29049137

[CR42] Schött U, Sjöstrand U, Thorén T, Berséus O (1985). Three percent dextran-60 as a plasma substitute in blood component therapy. I. An alternative in surgical blood loss replacement. Acta Anaesthesiol Scand.

[CR43] Schött U, Solomon C, Fries D, Bentzer P (2016). The endothelial glycocalyx and its disruption, protection and regeneration: a narrative review. Scand J Trauma Resusc Emerg Med.

[CR44] Statkevicius S, Bonnevier J, Bark BP, Larsson E, Öberg CM, Kannisto P, Tingstedt B, Bentzer P (2016). The importance of albumin infusion rate for plasma volume expansion following major abdominal surgery - AIR: study protocol for a randomised controlled trial. Trials.

[CR45] Stissing T, Dridi NP, Ostrowski SR, Bochsen L, Johansson PI (2011). The influence of low platelet count on whole blood aggregometry assessed by multiplate. Clin Appl Thromb Hemost.

[CR46] Thomas O, Rein H, Strandberg K, Schött U (2016). Coagulative safety of epidural catheters after major upper gastrointestinal surgery: advanced and routine coagulation analysis in 38 patients. Perioper Med (Lond).

[CR47] Treib J, Haass A, Pindur G (1997). Coagulation disorders caused by hydroxyethyl starch. Thromb Haemost.

[CR48] Vercueil A, Grocott MPW, Mythen MG (2005). Physiology, pharmacology, and rationale for colloid administration for the maintenance of effective hemodynamic stability in critically ill patients. Transfus Med Rev.

[CR49] Weisel JW, Litvinov RI (2013). Mechanisms of fibrin polymerization and clinical implications. Blood.

[CR50] Winstedt D, Hanna J, Schött U (2013). Albumin-induced coagulopathy is less severe and more effectively reversed with fibrinogen concentrate than is synthetic colloid-induced coagulopathy. Scand J Clin Lab Invest.

[CR51] Wyatt AR, Zammit NW, Wilson MR (2013). Acute phase proteins are major clients for the chaperone action of α_2_-macroglobulin in human plasma. Cell Stress Chaperones.

[CR52] Zdolsek HJ, Vegfors M, Lindahl TL, Törnquist T, Bortnik P, Hahn RG (2011). Hydroxyethyl starches and dextran during hip replacement surgery: effects on blood volume and coagulation. Acta Anaesthesiol Scand.

